# Forty-four-year longitudinal study of stroke incidence and risk factors – the Prospective Population Study of Women in Gothenburg

**DOI:** 10.1080/02813432.2022.2057040

**Published:** 2022-04-08

**Authors:** Ann Blomstrand, Christian Blomstrand, Magnus Hakeberg, Valter Sundh, Lauren Lissner, Cecilia Björkelund

**Affiliations:** a Primary Health Care, School of Public Health and Community Medicine, Institute of Medicine, Sahlgrenska Academy, University of Gothenburg, Gothenburg, Sweden; b Department of Clinical Neuroscience, Stroke Centre West, Institute of Neuroscience and Physiology, Sahlgrenska Academy, University of Gothenburg, Gothenburg, Sweden; c Institute of Odontology, Sahlgrenska Academy, University of Gothenburg, Gothenburg, Sweden; d Institute of Neuroscience and Physiology, Sahlgrenska Academy, University of Gothenburg, Gothenburg, Sweden; e Nutrition Epidemiology, School of Public Health and Community Medicine, Institute of Medicine, Sahlgrenska Academy, University of Gothenburg, Gothenburg, Sweden

**Keywords:** Women, stroke, risk factors, education, pre-eclampsia, oral health, atrial fibrillation, diabetes

## Abstract

**Objective:**

To assess stroke incidence over 44 years and association with risk factors. To study total stroke incidence at 60–82 years of age and risk factors.

**Design:**

Prospective population study.

**Setting:**

Gothenburg, Sweden, with ∼450,000 inhabitants.

**Subjects:**

A representative sample of a general population of women (1462 in total) in 5 age strata aged 38–60 years in 1968–1969 (the Population Study of Women in Gothenburg, PSWG) were followed up to the ages of 82–104 years in 2012. Further, analysis was also performed for the age interval 60–82 years.

**Main outcome measures:**

Incidence of total stroke (TS), ischaemic (IS), haemorrhagic (HS), non-specified (NS) and fatal (FS) strokes and association with baseline classic risk factors (such as hypertension, atrial fibrillation, low physical activity, diabetes, high waist-hip-ratio, hyperlipidaemia, smoking), low education, mental stress, pre-eclampsia and oral health as expressed by loss of teeth and bone score. Blood pressure in levels 1–3 according to modern guidelines. Associations with atrial fibrillation, diabetes and myocardial infarction shown in survival analyses. The five cohorts contributed to risk time data concerning associations with TS in the 60–82 age interval from the examination performed when they were 60.

**Results:**

Three hundred and thirty-seven (23%) women had a first-ever stroke, 64 (19%) fatal. TS was associated with physical inactivity, high triglycerides and low education in multivariable analysis. The main sub-type IS was associated with systolic blood pressure, physical inactivity and low education. Pre-eclampsia showed association with IS only in the univariable analysis. FS was associated with systolic blood pressure and smoking. During 60–82 years of age, having <20 teeth (HR 1.74, CI 1.25–2.42), diabetes (HR 2.28 CI 1.09–4.76), WHR (HR 1.29 per 0.1 units CI 1.01–1.63), systolic blood pressure (HR 1.11 per 10 units CI 1.04–1.18) and smoking (HR 1.57, CI 1.14–2.16), were associated with TS in the combined five cohorts.

**Conclusions:**

Several classic risk factors showed independent associations with stroke. Vulnerability factors as low education and oral health, reflected by loss of teeth, also showed association with stroke. All these factors are possible to target in primary care preventive interventions.

**Key Points**
- Stroke is a common disease and the risk of stroke is a key issue demanding preventive strategies in primary health care. The present prospective population study of women shows
- Out of 1460 women, almost a quarter got a stroke. The stroke incidence 60–82 years of age was rather stable between the first four age cohorts but somewhat lower in the latest cohort, born 1930.
- Hypertension, low physical activity, low education and high triglyceride levels but not cholesterol were associated with stroke in women.
- Low education and loss of teeth are vulnerability factors that should need particular attention.

## Introduction

Stroke is a leading cause of death and disability worldwide [1]. Incidence and mortality of stroke have shown a declining trend over time, driven by a decrease of ischaemic stroke in men [2]. Gender differences are reported, women being older at their first-ever stroke with higher stroke incidence above 85 years of age [3]. Modifiable risk factors explain around 90% of all acute stroke [4]. Health care systems and socioeconomic factors can influence stroke incidence [5,6]. Primary care has an important role to notice early unhealthy lifestyle patterns and risk factors for stroke and cardiovascular diseases and to manage health promotion strategies also with regard to social vulnerability [7–10]. Abdominal obesity has increased in middle-aged women as well as experience of mental stress [11].

Increased stroke risk associated with low education, particularly in women is largely explained by modifiable risk factors [12]. Negative lifestyle appears to increase the risk for stroke in people with lower socioeconomic position [13].

The prospective Population Study of Women in Gothenburg (PSWG) offers unique possibilities to follow stroke incidence in a defined female population followed since 1968 [14]. Changing diagnostic procedures, introduction of specialised stroke unit care and increased awareness of stroke can lead to bias in register-based studies when comparing hospital stroke diagnoses over time. Furthermore, determination of risk factors such as hypertension, diabetes and hyperlipidaemia has varied over time. Particular interest is to know more about important risk factors after middle age when stroke incidence increases. Socioeconomic position (SEP) for women has changed over the last decades. Long time follow-up also gives possibility to compare those in professional work versus not regarding association with stroke. Further, PSWG has followed data on oral health and impact on stroke risk.

Our primary aim was to study women’s incidence of first-ever stroke, specified according to validated main type, and association with classic risk factors as well as with less well-known factors as SEP, working situation and oral health over a 44-year period. Further aim was to compare incidence and risk factors for stroke amongst the different birth cohorts from 60 to 82 years of age when stroke incidence is increasing.

## Methods

### Study population

**Figure 1.**
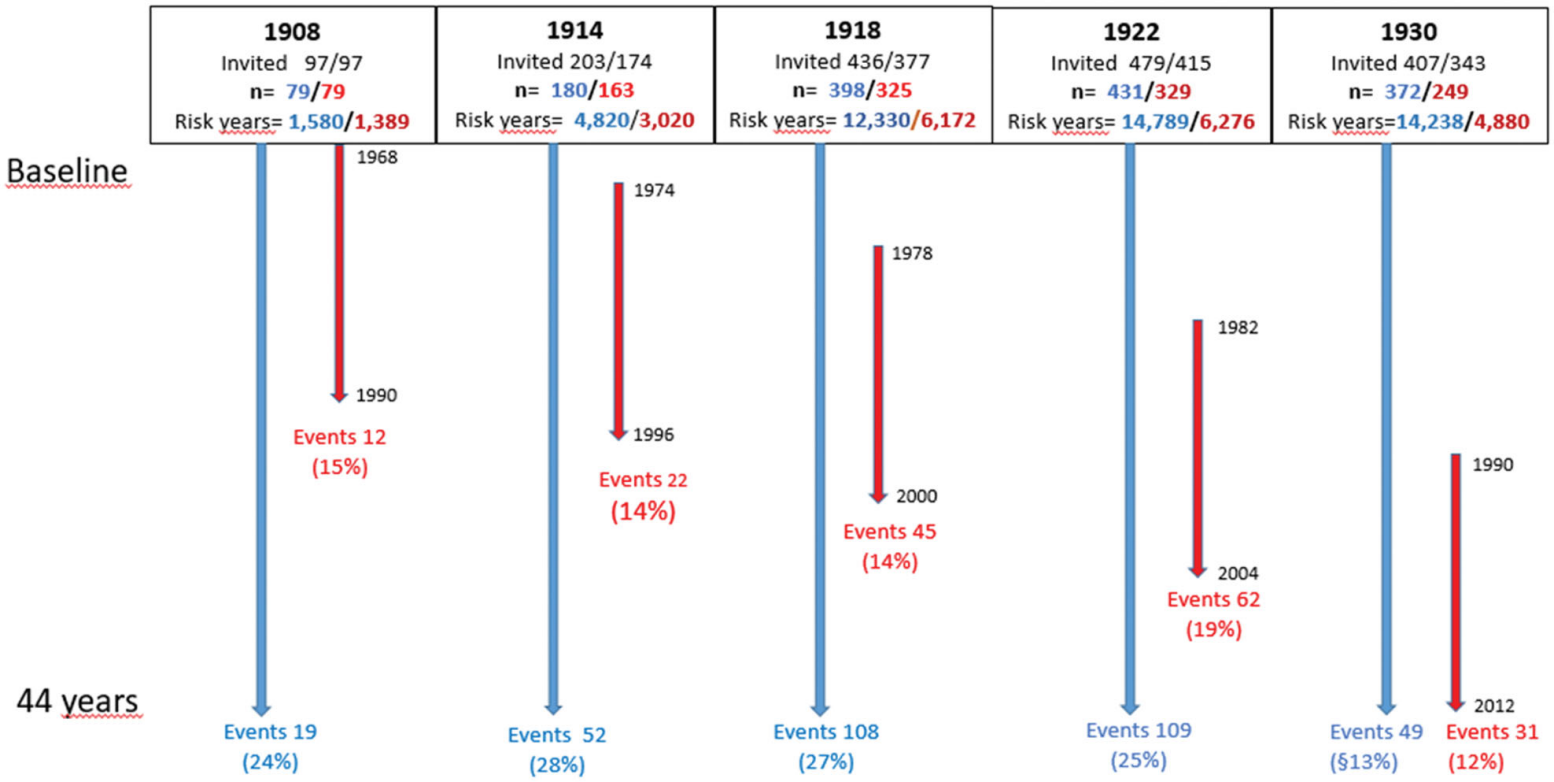
Five cohorts born 1908, 1914, 1918, 1922 and 1930 studied from baseline 1968–1969 during 44 years. Incidence of total stroke during 44 years (*n* = 337) (long arrows). Incidence of total stroke (*n* = 172) from 60 to 82 years of age (short arrows).

Five population-based cohorts born 1930, 1922, 1918, 1914 and 1908 were invited 1968–1969 and 1462 (90.1%) women participated. The outcomes for the present study were first-ever stroke and stroke mortality after 44 years. A further aim concerned stroke incidence in separate sub-cohorts at ages 60–82 years, by use of information from the follow-up examinations 1974–1975, 1980–1981 and 1992–1993 to define baseline data from the examinations closest to age 60, with range 58–62, this included 1145 women after exclusion of two individuals with stroke before age 60 (Figure 1). The same medical examinations and questionnaires were used at all follow-ups. Participation rates in survivors were 1974–1975 90%, 1980–1981 82% and 1992–1993 70%. Participation rate including non**-**survivors were 89%, 79% and 57%.

### Validation of stroke diagnoses

Classification of stroke according to the International Classification of Diseases (ICD) was previously described [15]. Hospital admissions in Sweden are registered in the National Patient Register (NPR). Endpoints were total stroke (TS) typed as fatal (FS) or non-fatal and further as ischaemic (IS) (*ICD-9 434, ICD-10 I63*), haemorrhagic (HS) (*ICD-9 431, ICD-10 I61*) and non-specified (NS). Subarachnoid haemorrhage was excluded. Medical records were scrutinised for all cases with unspecified or uncertain NPR stroke diagnoses (*ICD-9* 432, *ICD-10* I62 and *ICD-9* 436, *ICD-10* I64), as well as those with ICD codes (*ICD-9* 433, 434, 437, 438, *ICD-10* I65, I66, I67, I69). Further, codes for transient ischaemic attacks (TIA) (*ICD-9* 435, *ICD10*- G45) were scrutinised to reveal possible stroke.

### Potential risk factors for stroke

The PSWG included medical examinations, questionnaires, body mass index (BMI), waist-hip ratio (WHR), lipids with s-triglycerides, s-cholesterol total, (s-LDL, S-HDL only since 1992), blood pressure (BP) measures and ECG examinations. Hypertension at baseline, 1968–1969, was defined as ≥160/≥95 mm Hg and/or treatment [14]. For comparison with modern guidelines, we subdivided into four groups: a reference group (<140/<90), grade 1 (140–159 and/or 90–99), grade 2 (160–179 and/or 100–109) and grade 3 (≥180 and/or ≥110). Atrial fibrillation data were obtained from ECG examinations at each follow-up examination and hospital diagnoses. Myocardial infarction diagnoses were obtained by regular matching with National Patient Register and Mortality Register. Diabetes diagnoses were obtained *via* medical history interviews and fasting blood-glucose analyses. Smoking was dichotomised into current smokers and non-smokers. Self-reported intake of alcohol was assessed on a seven-level scale. As described in a recent study socioeconomic position (SEP) was defined from sociooccupational group classified as low, medium or high, based on reported occupation of spouse if available or own occupation and educational group [16]. Educational level was defined as low (elementary school only) and high (continued after elementary school) [14]. Self-reported leisure time physical activity and physical activity at work, respectively, was collected using a four-level scale. The levels were dichotomised as mostly physically inactive (level 1) and activities at least 4 h a week (level 2). Professional work was indicated as ‘yes’ or ‘no’. Self-perceived mental stress was evaluated on an ordinal scale from 0 to 5: 0 = no stress and 5 = continuous stress in the past 5 years and dichotomised such that >3 represented permanent stress.

Pre-eclampsia was based on a medical history questionnaire with eight questions concerning high blood pressure, albuminuria and general swelling. Oral health was based on dental examinations with number of teeth and level of periodontitis. The examination was carried out, per individual, by means of a clinical and radiographic examination (panoramic radiograph). The variable number of teeth was dichotomised with cut off at <20 teeth. Level of periodontitis was assessed from the radiographs by using a Schei ruler to measure bone loss adjacent to teeth by dividing the bone loss into five equal distances relative to the length of the roots of the teeth (1 = no bone loss; 5 = extreme bone loss). Accordingly, the level of periodontitis was estimated as a mean score bone loss [17].

### Statistical methods

Stroke risk was primarily analysed using all participants (*n* = 1459) during the whole follow-up 1968–2012, second stroke risk was analysed during the time period the different cohorts were aged 60–82 years. The primary analysis includes all available follow-up information on stroke incidence; in the secondary the comparison is restricted to the age interval when all five cohorts contribute data on stoke incidence (Tables 1a and 1b).

**Table 1a.** Data on stroke incidence for five birth year cohorts based on all 1968–2012 follow-up data.

Birth year	*N*	Stroke events	Proportion	Risk time years	Risk per 1000 years
1908	79	19	24.05%	1580	12.02
1914	179	52	29.05%	48,201	10.79
1918	398	108	27.14%	12,330	8.76
1922	431	109	25.29%	14,789	7.37
1930	372	49	13.17%	14,238	3.44
Total	1459	337	23.10%	47,758	7.06

Risk factors for stroke incidence were tested in Cox Proportional Hazard models and the results are presented as Hazard Ratios with 95% confidence intervals. Initially models were calculated for each potential risk factor adjusted only for age. Risk time models were also calculated from Poisson models with risk time divided into small independent time intervals with the stroke event coded as a 0–1 outcome in the appropriate time interval. These models give asymptotically the same parameter estimates as Cox models, but are in same situations easier to use.

The age intervals from baseline in 1968–1969 to end of follow-up in 2012 for the five cohorts are 60–104, 54–98, 50–94, 46–90 and 38–82 years.

Analysis of the full follow-up 1968–2012 used baseline data from 1968 to 1969 examination. The analysis limited to follow-up age 60–82 used data from the examination closest to each individual’s 60th birthday. Thus, this sample is limited to the individuals participating in a ‘60-year’ examination and were not classified as stroke cases before age 60. Birth cohort 1918 was measured nominally at age 58 and birth cohorts 1922 and 1930 at age 62, and cohorts 1908 and 1914 at age 60. However, for all persons risk time is defined to start the year they become 60 years old and ends the year they become 82.

Basic description of follow-up data from 1968 to 2012 and from age 60 to 82 are given in Tables 1a and 1b with number of total stroke incidence, number of risk years, rate per 1000 years in the separate cohorts and relative risk rate compared to the oldest cohort. The data for the 60–82 years follow-up are also presented with cohort 1908 − 1922 combined and compared to the 1930 cohort.

Additional adjustments included hypertension, BMI, WHR, smoking, physical activity, cholesterol, triglycerides, mental stress, medical history of pre-eclampsia, educational level and number of teeth. For age 60–82 having full-time or part-time work (no/yes) was also included. In addition, survival time 1968–2012 free from stroke was calculated for diabetes, myocardial infarction (MI), atrial fibrillation (AF) and hypertension at baseline. Interaction tests were performed to examine if the effect of some risk factors for stroke differed markedly between cohorts by adding an interaction term to the model defined as the product of two main effects in the model, e.g. low education × cohort.

## Results

### Stroke incidence – 44-year follow-up

Out of 1460 women, 337 (23%), had a first-ever stroke during the 44-year follow-up distributed as follows: 262 (78%) IS, 39 (11%) HS, 38 (11%) NS. Fatal first-ever strokes constituted 64 cases (19%). NS were through validation minimised to 11% from 19%. Crude incidence was 7.0 per 1000 person years, and, standardised to the female population in Gothenburg the year 2000, it was 6.2. Table 1a shows all data on stroke incidence 1968–2012. The stroke risk is lower in each later cohort due to the difference in age distribution during follow-up (fewer risk years at older ages in the later cohorts).

Table 1b shows the corresponding figures for the follow-up from 60 to 82 years for all cohorts, allowing a direct comparison of cohort difference in stroke risk. In cohorts 1908 − 1922 incidence rate (IR) per 1000 years was 8.4 and in cohort 1930 it was 6.4 with relative IR 0.759 (95% CI = 0.50–1.13). The difference between the four cohorts 1908–1922 was marginal, without any significant trend over birth years (all IRs in the range 7.3–9.9), whilst in a basic regression model there was a 38% higher risk in cohorts 1908–1922 (95% CI for HR = 0.93–1.03) compared to cohort 1930.

**Table 1b.** Data on stroke incidence for five birth year cohorts in age interval 60–82.

Birth year	*N*	Stroke events	Proportion	Risk time years	Risk per 1000 years	Incidence ratio	Incidence ratio 95% CI	2-side Prob.
1908	79	12	15.2%	1389	8.6	1.000	Ref. grp.	
1914	163	22	13.5%	3021	7.3	0.843	0.40 − 12.87	0.71
1918	325	45	13.8%	6172	7.3	0.844	0.44 − 1.75	0.61
1922	329	62	18.8%	6276	9.9	1.143	0.61 − 2.33	0.76
1930	249	31	12.4%	4880	6.4	0.735	0.38 − 1.57	0.36
Total	1145	172	15.0%	21,737	7.9			

### Potential baseline risk factors for stroke in 44-year follow-up

Table 2 shows univariable age-adjusted HRs of risk factors for stroke in the sub groups. All variables except cholesterol, mental stress and bone score showed significant associations with IS and TS. Pre-eclampsia was associated with IS. FS was associated with BP, WHR, smoking, physical inactivity, low education and teeth <20. Socioeconomic position (SEP) did not show association with stroke.

**Table 2.** Risk factors for stroke at baseline (1968).

	Ischaemic		Haemorrhagic		Total		Fatal stroke	
	HR	95%CI	HR	95%CI	HR	95%CI	HR	95%CI
Variable univariable model								
Systolic blood pressure per 10 mm Hg	**1.14**	**1.08 − 1.21**	**1.17**	**1.02 − 1.35**	**1.14**	**1.07 − 1.21**	**1.26**	**1.14 − 1.39**
Diastolic blood pressure per 10 mm Hg	**1.22**	**1.09 − 1.37**	1.27	0.95 − 1.70	**1.22**	**1.08 − 1.36**	**1.45**	**1.18 − 1.77**
Hypertension	**1.91**	**1.44 − 2.54**	1.69	0.82 − 3.50	**1.89**	**1.42 − 2.50**	**2.52**	**1.50 − 4.24**
BMI	**1.07**	**1.04 − 1.10**	1.00	0.92 − 1.10	**1.07**	**1.04 − 1.11**	1.05	0.99 − 1.12
WHR per 0.1 units	**1.56**	**1.24 − 1.97**	1.16	0.60 − 2.22	**1.59**	**1.26 − 2.01**	**2.16**	**1.18 − 3.28**
Smoking	**1.29**	**1.00 − 1.65**	1.49	0.78 − 2.86	**1.33**	**1.06 − 1.66**	**2.13**	**1.29 − 3.51**
Physical inactivity	**1.47**	**1.08 − 1.99**	1.71	0.81 − 3.61	**1.54**	**1.18 − 2.01**	**1.78**	**1.00 − 3.18**
Cholesterol (mmol/l)	1.07	0.98 − 1.17	0.75	0.54 − 1.04	1.04	0.95 − 1.13	1.08	0.91 − 1.28
Triglycerides (mmol/l)	**1.17**	**1.08 − 1.27**	0.88	0.58 − 1.35	**1.18**	**1.09 − 1.27**	1.17	0.999 − 1.37
Mental stress	0.93	0.71 − 1.21	0.98	0.50 − 1.92	0.93	0.74 − 1.17	0.90	0.53 − 1.51
Low education	**1.61**	**1.19 − 2.14**	1.08	0.53 − 2.19	**1.41**	**1.10 − 1.81**	**1.92**	**1.03 − 3.61**
Pre-eclampsia	**1.46**	**1.01 − 1.88**	1.47	0.68 − 3.20	1.35	0.99 − 1.84	1.14	0.59 − 2.18
Teeth < 20	**1.46**	**1.14 − 1.87**	1.65	0.86 − 3.13	**1.46**	**1.14 − 1.87**	**2.59**	**1.55 − 4.35**
Bone score	1.11	0.86 − 1.44	**1.96**	**1.13 − 3.41**	1.09	0.84 − 1.41	**1.61**	**1.01 − 2.58**
Variable multivariable model								
Systolic blood pressure per 10 mm Hg	**1.07**	**1.01 − 1.14**	1.11	0.95 − 1.30	1.06	0.997 − 1.13	**1.15**	**1.03 − 1.28**
BMI	1.04	0.99 − 1.08	1.00	0.90 − 1.11	1.04	0.997 − 1.08	0.99	0.91 − 1.07
WHR per 0.1 units	1.08	0.83 − 1.42	0.95	0.44 − 2.05	1.10	0.84 − 1.45	1.59	0.92 − 2.76
Smoking	1.21	0.91 − 1.60	1.55	0.75 − 3.19	1.17	0.88 − 1.55	**2.06**	**1.20 − 3.53**
Physical inactivity	**1.42**	**1.03 − 1.95**	1.74	0.80 − 3.75	**1.42**	**1.03 − 1.95**	1.51	0.83 − 2.76
Triglycerides (mmol/l)	1.11	0.999 − 1.24	0.75	0.44 − 1.28	**1.12**	**1.003 − 1.24**	0.99	0.74 − 1.33
Low education	**1.42**	**1.04 − 1.94**	0.96	0.46 − 2.01	**1.42**	**1.04 − 1.93**	1.50	0.77 − 2.89
Pre-eclampsia	1.31	0.95 − 1.80	1.41	0.61 − 3.27	1.30	0.95 − 1.79	1.02	0.51 − 2.04
Teeth < 20	1.08	0.82 − 1.42	1.44	0.89 − 2.99	1.08	0.82 − 1.43	1.64	0.94 − 2.89

Cox regression analysis. Univariable models age-adjusted analysis (upper part), and multivariable analysis (lower part). HR with 95% CI for stroke compared with women free from stroke. 44-year follow-up.

Bold typeface, statistically significant HR at *p* ≤ .05.

In multivariable Cox regression analyses significant associations were found between IS and systolic blood pressure, physical inactivity and low education (Table 2). Triglycerides, physical inactivity and low education were significantly associated with TS, and systolic blood pressure and smoking with FS. The analysis of association between risk of total stroke and BP levels showed significant association with systolic hypertension grade 2: HR 2.3 (CI 1.60–3.39) and grade 3: HR 3.17 (CI 2.01–5.00) as well as diastolic hypertension grade 2: HR 1.70 (CI 1.16–2.50) and grade 3: HR 2.30 (CI 1.33–3.95).

### Survival analysis concerning free from stroke for cases with diabetes mellitus, atrial fibrillation and myocardial infarction

**Figure 2.**
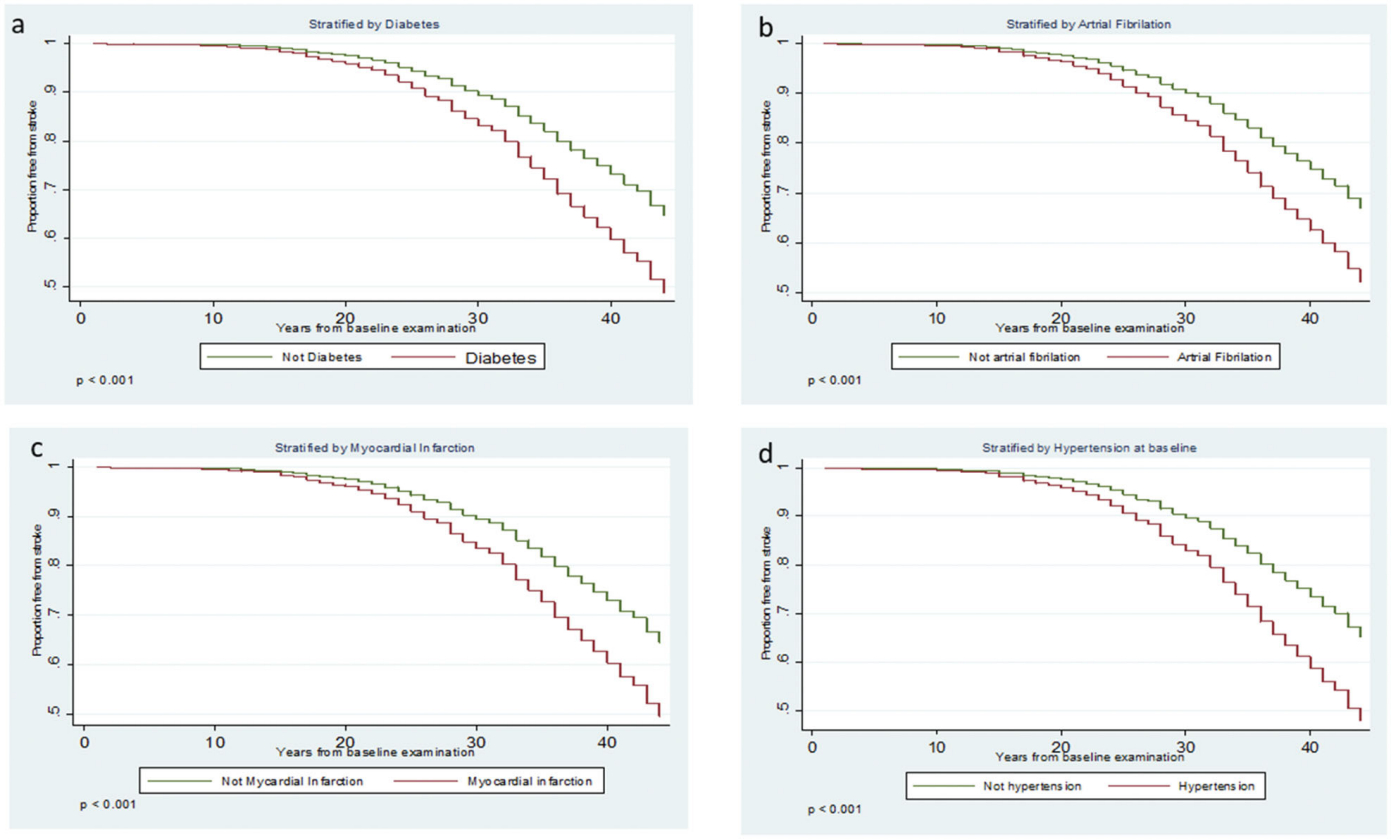
Forty-four year survival curves based on the Cox regression analysis of stroke with diabetes (a), atrial fibrillation (b), myocardial infarction (c) and baseline hypertension (d).

The 44-year survival analysis showed significantly increased time free from stroke in individuals without concurrent diabetes (*p* < .001), atrial fibrillation (*p* < .001), myocardial infarction (*p* < .001) and hypertension at baseline (*p* < .001) (Figure 2).

### Risk factors for total stroke from 60 to 82 years of age

#### Analysis for all five cohorts (1908–1930), the four earliest (1908–1922), the latest (1930)

Table 3 shows multivariable Cox regression analysis to compare associations with risk factors for TS in the combined five cohorts, and the four earliest and the latest cohort separated. Strong association with teeth <20 was seen in all three models. WHR was significantly associated with stroke in the combined cohorts (1908 − 1930) as was also systolic blood pressure, diabetes, smoking and having less than 20 teeth.

**Table 3.** Multivariable Cox regression analysis.

Variable	Syst blood pressure		Diabetes		WHR		Teeth <20		Smoking	
Cohorts	HR	95%CI	HR	95%CI	HR	95%CI	HR	95%CI	HR	95%CI
1908–1930	**1.11**	**1.04–1.18**	**2.28**	**1.09–4.76**	**1.29**	**1.01–1.63**	**1.74**	**1.25–2.42**	**1.57**	**1.14–2.16**
1908–1922	**1.12**	**1.04–1.20**	**2.33**	**1.00–5.46**	1.23	0.95–1.59	**1.64**	**1.12–2.41**	**1.72**	**1.21–2.47**
1930	1.05	0.90–1.22	1.39	0.30–6.47	1.60	0.72–3.54	**2.23**	**1.03–4.80**	0.92	0.42–2.00

Significant predictors after stepwise selection for total stroke in the five cohorts (1908–1930), chosen from the significant predictors of total stroke in the 44-year follow-up in Table 2 univariable analysis. Results separately in the cohorts (1908–1930), (1908–1922) and 1930, from 60 to 82 years of age. HR with 95% CI for stroke compared with women free from stroke.

Bold typeface, statistically significant HR.

### Professional work versus no work and risk for total stroke (60–82 years of age)

In the cohorts 1908–1922, there was a lower risk of stroke for those with professional work (*n* = 609) compared to those not working (*n* = 512), HR 0.92 (CI 0.51–0.999) (age-adjusted model). In cohort, 1930 no difference was observed, HR 1.12 (CI 0.55 − 2.26).

## Discussion

From a 44-year perspective, almost 25% of participants in the PSWG got a stroke and the main risk factors were hypertension, high triglycerides, low education and physical inactivity. Stroke fatality was associated with systolic blood pressure and smoking. Between 60 and 82 years in the five cohorts significant risk factors were systolic blood pressure, diabetes, WHR, smoking and oral health as reflected by number of teeth were associated with stroke.

The population was well defined with a long follow-up time, high initial participation rate and a low drop-out rate. Endpoint validation through careful medical record examination, including CT examinations, decreased the number of incorrect diagnoses. CT has been performed in Gothenburg as routine in acute stroke from around 1980. Still the number of haemorrhagic strokes was too low to find significant risk factor associations. Stroke fatality reflects the most severe strokes.

This long-term study includes comparison between five age cohorts reflecting different phases in women’s life. Further, the broad spectrum of parameters including oral health and social factors contributes important knowledge. Observations from ages 60 to 82 with risk data from examinations around 60 years of age show risk factors for stroke in a phase of life before the steep increase in stroke and severe comorbidities amongst elderly women.

One limitation is that the number of participants is limited and conclusions regarding other ethnic groups or rural populations cannot be drawn. Further, the low total number of haemorrhagic strokes only revealed statistically significant results for systolic blood pressure. Another limitation is that socioeconomic factors important now are not always congruent with corresponding factors half a century ago. This may lead to underestimation of association between socioeconomic situation and stroke. We cannot rule out that socioeconomic factors might have influenced seeking hospital care pattern. Another limitation is that we have not studied risk factor load over time with different analyses such as in an earlier study on men [18]. Further, we cannot rule out that repeated examinations over long time may have affected the women’s awareness of risk factors. Another limitation is that changing panoramas of diseases and treatments can infer some bias through increasing survival after severe diseases, which might have led to a healthier population.

The incidence figures were standardised using the Gothenburg female population in year 2000. Our rate of 7.0 per 1000 person years is higher than that in a recent report from southern Sweden [19], possibly due to our strict population-based longitudinal design with long-term follow-up. Further, the southern Sweden study showed decreased stroke incidence amongst elderly during the past two decades whilst our study includes stroke during 44 years until 2012. Comparing the five cohorts, incidence was lowest in the latest cohort born 1930. Part of the lower risk in the latest born cohort might be connected to better control of hypertension the last decades. The population-based Dijon Stroke study with 25 years of follow-up also showed lower incidence rates [20] compared to our study.

A meta-analysis showed an association between SEP and stroke, partly explained by classic risk factors [21]. Social factors have been reported to have a particularly strong impact on stroke incidence in women [22]. Although women’s situation has changed in Swedish society, awareness of social inequities is still needed [23]. A meta-analysis regarding sex differences in socioeconomic status showed that associations with coronary heart disease and cerebrovascular disease were stronger in women [5]. Another review highlights several sex differences regarding stroke and risk factors [6]. Another cohort study in Swedish women showed an inverse association between risk of stroke and years of education [24] compatible with our study. In a population study of men born 1913 followed for 48 years, low education was strongly associated with stroke [18]. Our study showed a strong association between education and stroke whilst SEP did not show any association. However, the present baseline data were from another societal situation for women. An earlier Swedish study showed that low education persisted as risk also in higher ages [25].

It is interesting to note the strong association with stroke between 60 and 82 years of age that was shown for reduced number of teeth. This robust association may partly be caused by social factors, secondary effects of periodontal disease and inflammatory responses. The risk associated with poor oral health [26], showed association between tooth loss and cardiovascular disease irrespective of socioeconomic status.

Knowledge about the association between poor oral health and systemic disease including stroke has increased during the last decades [27,28]. Population-based studies have found a significant association between periodontal disease and cardiovascular disease, stroke and all-cause mortality [29]. The REGARDS study showed associations between tooth loss, inflammatory markers and prevalence of stroke [30]. A review reported that periodontitis and tooth loss were associated with stroke [28]. Number of missing teeth can be an effect of health-related behaviour [27]. Thus, tooth loss and periodontal disease may be indicative of several shared risk factors together with diseases such as stroke.

Pre-eclampsia was associated with higher risk for stroke though not persisting in the multivariable analyses. Increased stroke risk in acute phases of pre-eclampsia is well known and is a risk factor for later stroke [31]. The need of careful and long-term follow-up regarding all risk factors have been pointed out in a recent review [8], as well as of primary care preventive actions.

Diabetes before 60 years of age increased the risk for total stroke. Documented atrial fibrillation was recruited from electrocardiograms at defined time points and from registers, which probably underestimates this major risk factor since additional intermittent episodes can have occurred. In a meta-analysis 2016, atrial fibrillation was a significantly stronger stroke risk factor for women compared to men [32].

As mentioned previously, risk factors for cardiovascular disease have been described in many male cohorts, some of which are similar to findings in women, whilst other can differ. In a male population born 1913 in Gothenburg followed for 48 years, a 20% stroke incidence amongst 854 men was observed i.e. similar to our study [18]. Total and ischaemic stroke risk factors in men were comparable to those in women except for triglyceride levels, which were only associated with stroke in women. In a systematic review [33], cholesterol appeared as a strong risk factor for coronary heart disease particularly in men but not for stroke in either sexes. Our study is also compatible with the INTERSTROKE case-control study [34], which showed that high cholesterol was not associated with ischaemic stroke. A recent study [35] concluded that low LDL and triglyceride levels were associated with increased risk for haemorrhagic stroke in women. Our study lacked power to reveal differences concerning haemorrhagic stroke.

Mental stress was not associated with stroke in the present study or in the male Gothenburg population study [18].

## Conclusion

Almost a quarter of the women suffered a first-ever stroke over 44 years, and 15% between 60 and 82 years of age. Lifestyle factors were associated with the risk for stroke as well as high triglyceride levels, but high cholesterol did not show any association with stroke in women. Poor dental health and low education can be indicators of low socioeconomic position and increased risk of later stroke. Collaboration between primary care and dental health care for risk cases might be a valuable issue. Long-term follow-up of borderline or high blood pressure and management of diabetes appears urgent. Primary health care has an important role in primary and secondary prevention of the common stroke disease.

## Ethics approval

The Population Study of Women in Gothenburg was approved by the Ethics Committee of Gothenburg University, Dnr 179-92 S 208-01 2000, Ö 402-99, T331-14.
